# The increase in bladder carcinoma cell population induced by the free beta subunit of human chorionic gonadotrophin is a result of an anti-apoptosis effect and not cell proliferation

**DOI:** 10.1054/bjoc.2000.1177

**Published:** 2000-04-03

**Authors:** S A Butler, M S Ikram, S Mathieu, R K Iles

**Affiliations:** Williamson Laboratory, Department of Obstetrics and Gynecology, St Bartholomew's and the Royal London School of Medicine and Dentistry, London, EC1A 7BE, UK

**Keywords:** free hCGβ, cystine knot growth factor, apoptosis, bladder cancer

## Abstract

Ectopic production of free beta human chorionic gonadotrophin (hCGβ) by bladder carcinoma is well described and occurs in approximately 35% of cases. hCGβ secreting tumours are more aggressive, radioresistant and have a greater propensity to metastasize. We proposed that the ectopic production of hCGβ was contributing in an autocrine fashion to the radioresistance and metastatic potential of such secreting tumours. Though we demonstrated that the addition of hCGβ to the culture media of bladder, cervical and endometrial carcinoma cell lines brought about an increase in cell populations this was not accompanied by a significant increase in the rate of replication. Since a cell population size is a balance of mitosis and mortality, we proposed that hCGβ was inhibiting apoptosis. Here we have demonstrated that following incubation with recombinant hCGβ, bladder carcinoma cells refrain from undergoing apoptosis. Quantitation of apoptotic bodies was carried out by immunoassay and corrected to cell number as determined by MTT assay. In each cell line, addition of hCGβ reduced the number of apoptotic bodies dose-dependently, indicating a diminished apoptotic rate. Furthermore, TGFβ1-induced apoptosis could be dose-dependently inhibited by co-incubation with hCGβ. We propose, therefore, that such a decline in apoptosis may account for the cell population increase previously reported. It may also explain the radioresistance and aggressive nature of hCGβ-secreting tumours and the poor prognosis associated therein. © 2000 Cancer Research Campaign


*British Journal of Cancer* (2000) **82**(9), 1553–1556
© 2000 Cancer Research Campaign
DOI: 10.1054/ bjoc.2000.1177, available online at http://www.idealibrary.com on IDEAL®

### The increase in bladder carcinoma cell population induced by the free beta subunit of human chorionic gonadotrophin is a result of an anti-apoptosis effect and not cell proliferation

**SA Butler, MS Ikram, S Mathieu and RK Iles**

Williamson Laboratory, Department of Obstetrics and Gynecology, St Bartholomew's and the Royal London School of Medicine and Dentistry, London EC1A 7BE, UK

**Summary** Ectopic production of free beta human chorionic gonadotrophin (hCGβ) by bladder carcinoma is well described and occurs in approximately 35% of cases. hCGβ secreting tumours are more aggressive, radioresistant and have a greater propensity to metastasize. We proposed that the ectopic production of hCGβ was contributing in an autocrine fashion to the radioresistance and metastatic potential of such secreting tumours. Though we demonstrated that the addition of hCGβ to the culture media of bladder, cervical and endometrial carcinoma cell lines brought about an increase in cell populations this was not accompanied by a significant increase in the rate of replication. Since a cell population size is a balance of mitosis and mortality, we proposed that hCGβ was inhibiting apoptosis. Here we have demonstrated that following incubation with recombinant hCGβ, bladder carcinoma cells refrain from undergoing apoptosis. Quantitation of apoptotic bodies was carried out by immunoassay and corrected to cell number as determined by MTT assay. In each cell line, addition of hCGβ reduced the number of apoptotic bodies dose-dependently, indicating a diminished apoptotic rate. Furthermore, TGFβ1-induced apoptosis could be dose-dependently inhibited by co-incubation with hCGβ. We propose, therefore, that such a decline in apoptosis may account for the cell population increase previously reported. It may also explain the radioresistance and aggressive nature of hCGβ-secreting tumours and the poor prognosis associated therein. © 2000 Cancer Research Campaign

**Keywords**: free hCGβ, cystine knot growth factor, apoptosis, bladder cancer

The beta subunit of human chorionic gonadotrophin (hCGβ) confers the structural and functional identity of the biologically active glycoprotein heterodimer of hCG. The alpha subunit is common to all members of the glycoprotein hormone family, which includes thyroid stimulating hormone (TSH), follicle stimulating hormone (FSH) and luteinizing hormone (LH). Only the intact α-β arrangement is hormonally functional; the free subunits themselves do not exhibit gonadotrophic or thyrotrophic activity, and the beta subunit alone is not sufficient to stimulate the LH/hCG receptor (Pierce and Parsons, 1981). Therefore, any growth factor activity exhibited is generally regarded to be occurring via a novel pathway.

The ectopic production of free hCGβ – in the absence of the alpha subunit – is a well-recognized phenomenon in many epithelial tumours (Braunstein, 1983; Iles and Chard, 1989). It was originally regarded as an epiphenomenon, with no biological significance given the absence of hCGα and a functional heterodimeric structure. More recently, however, it has been shown that the beta subunit may have its own unique functions. Lunardi-Iskander et al (1995) and Gillott et al (1996) have independently shown growth inhibitory and growth stimulatory effects of free hCGβ. Elucidation of the crystal structure of hCG revealed surprising topological similarities with the known 'cystine knot' growth factors – transforming growth factor beta (TGF-β), platelet derived growth factor beta (PDGFβ) and nerve cell growth factor (NGF). In particular, hCGβ and its urinary breakdown product, the hCGβ core fragment (hCGβcf), bear a striking similarity to TGFβ and PDGFβ respectively (Lapthorn et al, 1994). Furthermore, the growth factor family also share a common feature of only functioning as hetero- and/or homodimers and investigations into hCGβ as a cystine knot growth factor have shown that, along with the established heterodimeric structure of the hormone hCG, hCGβ and hCGβcf also form homodimers (Butler et al, 1999; Iles et al, 1999).

These surprising new findings support the hypothesis that the ectopic production of hCGβ by epithelial tumours is not an epiphenomenon, but rather a significant release of a possible autocrine/paracrine growth factor which is contributing directly to the poor prognosis of these tumours. Further studies into the stimulation of bladder carcinoma cells with recombinant hCGβ continue to bring about an increase in cell number when quantified by 3-[4,5-dimethylthiazol-2-yl]-2,5-diphenyl tetrazolium bromide (MTT) reduction. However, cell replication – as quantified by thymidine incorporation (unpublished data) – is not consistent with the same increase in population, suggesting that the hCGβ is not, in fact, increasing the growth rate, but rather is slowing the rate of apoptosis. Here we have investigated apoptosis in bladder carcinoma cell lines in response to hCGβ stimulation.

*Received 14 July 1999*
*Revised 6 January 2000*
*Accepted 17 January 2000*

*Correspondence to:* RK Iles

### MATERIALS AND METHODS

### Growth factors

Recombinant hCGβ and recombinant TGFβ1 (Sigma, Poole, UK) were reconstituted according to the manufacturer's recommendations. Aliquots of reconstituted proteins were diluted to working concentrations in RPMI-1640 cell culture medium (Sigma) containing 10% fetal calf serum (FCS) and 1% antibiotic/antimycotic solution (Gibco-BRL, Life Technologies Ltd, Paisley, UK).

### Cell lines

Bladder carcinoma cell lines T24 (Dr J Masters, Institute of Urology, London, UK), SCaBER, J82, 5637 and RT112 (American type Culture Collection, Rockville, MD, USA) were used in the incubation studies. T24 has been shown to be the most sensitive to the addition of hCGβ believed to be due to the fact that it produces no endogenous hCGβ of its own (Gillott et al, 1996). SCaBER and RT112 are high level hCGβ producers and 5637 and J82 low level hCGβ producers (Iles and Chard, 1989).

### MTT assay

A variation on the MTT assay (Mosman, 1983; Twentyman and Luscome, 1987) was used to determine cell numbers following an incubation with a growth factor or combination of growth factors.

Ninety-six-well plates (Flat-bottomed, Nunc, Gibco-Life Technologies) were seeded with $5 \times 10^4$ cells per well, in RPMI-1640/10% FCS/1% antibiotic antimycotic and incubated at 37°C in the presence of 5% carbon dioxide for 24 h. The media were then replaced with new media containing the growth factor material (0–400 pmol $ml^{-1}$), in replicates of 6 and incubated as before, for a period of 48 h. Control plates were treated with the same concentrations of TGF-β1, a known inducer of epithelial cell apoptosis and a member of the same family of growth factors as hCGβ. In the co-incubation experiments apoptosis was induced by incubation in 100 pmol $ml^{-1}$ TGF-β1 in addition to increasing concentrations (0–400 pmol $ml^{-1}$) of hCGβ. Following this, the cells were subjected to MTT assay. Media was again replaced with RPMI without phenol red and further incubated for a period of 1 h, upon which 20 μl of sterile filtered MTT (Sigma) (5 mg $ml^{-1}$ in phosphate-buffered saline) was added to each well. After a 3-h incubation all solutions were removed from the cells and replaced with 200 μl of dimethylsulphoxide (DMSO, Sigma, UK) and the formizan crystals allowed to dissolve. Each well was then read for absorbance at 570 nm in a Microplate autoreader (Bio-Tek instruments).

Prior to MTT assay an aliquot of incubation media was removed from each well and assayed for the presence of mono- and oligonucleosomes using the Boehringer Mannheim apoptosis cell death detection enzyme-linked immunosorbent assay. Nucleosome concentration was then determined from the spectrophotometric data and corrected to cell number as subsequently determined by tetrazolium salt reduction.

### RESULTS

Absorbance data were corrected to percentage change from untreated control. The percentage change in cell number and percentage change in nucleosome concentration clearly indicate a rise in cell number accompanied by a corresponding fall in nucleosome concentration in response to hCGβ. Conversely TGF-β1 brings about a dose-dependent fall in cell number and rise in nucleosome concentration (Figure 1 A, B). Figure 2 indicates the results of the co-incubation study, a sharp rise in the percentage number of nucleosomes (and hence increase in apoptosis) following incubation with 100 pmol $ml^{-1}$ TGF-β1. The effect gradually diminishes to below 100% as the concentration of hCGβ increases from 0 to 400 pmol $ml^{-1}$, despite the continued presence of TGFβ1.

### DISCUSSION

The ectopic production of free hCGβ is a well-recognized phenomenon in many epithelial tumours (Cole et al, 1988). It is now apparent that this event may significantly affect tumour development given the growth effects recently described (Lunardi-Iskander et al, 1995; Gillott et al, 1996). It is accepted that free hCGβ is unable to activate the LH/hCG receptor and stimulate the subsequent second messengers. Therefore the recently reported growth factor activities of hCGβ are assumed to proceed via novel, and as yet unidentified, pathways. Following the elucidation of hCG's crystal structure, its subunits were grouped with the family of cystine knot growth factors which includes TGF, PDGF and NGF (Lapthorn et al, 1994). The topological similarities observed – particularly the presence of the central cystine knot – suggest a common growth factor function. This concept has recently been reinforced by data which clearly indicate the presence of hCGβ homodimers that, like TGF, PDGF and NGF, may be required to bring about cellular growth responses.

In this study we have reconfirmed the apparent growth stimulation of bladder carcinoma cells by hCGβ in comparison to the effect brought about by TGF-β1, a cystine knot growth factor and known inducer of epithelial apoptosis (Sporn et al, 1986; Sun and Davies, 1995). Figure 1A shows the contrasting effects of TGF-β1 and hCGβ on cell number in five bladder carcinoma cell lines. The change in apoptosis in response to hCGβ or TGF-β1 can be seen in Figure 1B and could certainly account for the difference in cell number observed in the previous figure (Figure 1A). We propose that the increase in cell number brought about by hCGβ could be solely accounted for by a reduction in apoptosis. The sharp rise in cell numbers in response to low concentrations of hCGβ followed by a plateau indicates that the molecule may be acting in an inhibitory fashion given that there is a sudden change in percentage cell number at a given hCGβ concentration and not a gradual dose-dependent response. The results following co-incubation of 5637 cells with constant TGF-β1 and increasing hCGβ indicate that hCGβ can reverse the apoptotic effect brought about by the addition of TGF-β1. An increase in nucleosome concentration in the presence of 100 pmol $ml^{-1}$ TGF-β1 is consistent with that seen for 5637 in Figure 1B. However, as the hCGβ concentration increases the number of nucleosomes reduces to below that seen in the negative control despite the presence of the TGF-β1.

In light of the particular topological similarities between hCGβ and TGF-β and the opposing nature of their effect on epithelial cells, it could be suggested that hCGβ may be interacting with the TGF-β receptor complex. Interactions between the cystine knot growth factors is not uncommon, and TGF-β and PDGF in particular, cooperate to bring about their designated responses. The hCGβ homodimer recently described could be such a candidate for

 © 2000 Cancer Research Campaign

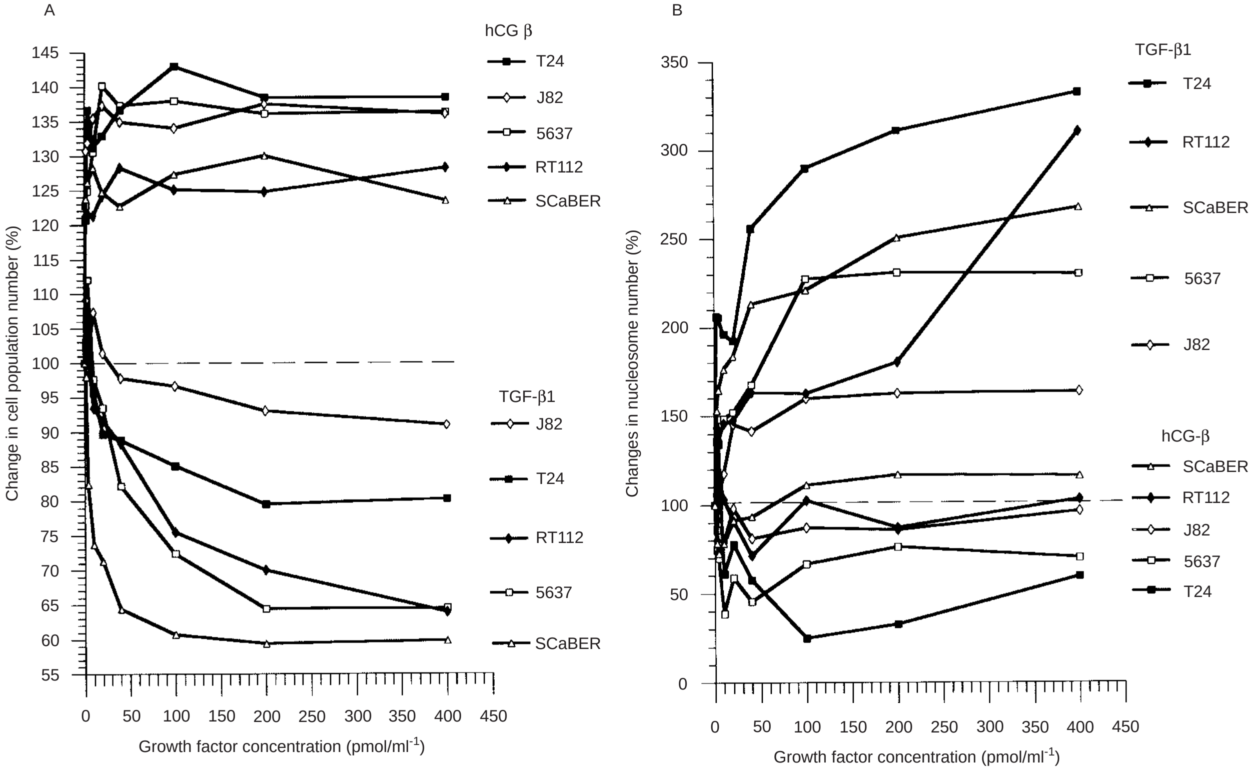

**Figure 1** (**A**) The percentage change in cell number from the control (100%, and indicated by the dashed line) as a result of incubation with hCGβ (top) and TGF-β1 (bottom) on the five different bladder cancer cell lines. (**B**) The percentage change in nucleosome concentration from the control (100%, and indicated by the dashed line) as a result of incubation with hCGβ (bottom) and TGF-β1 (top) on the five different bladder cancer cell lines

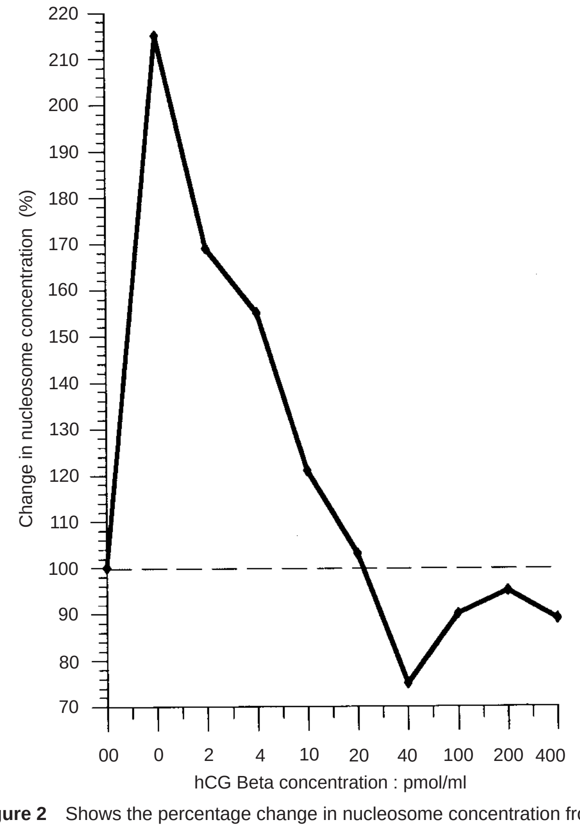

**Figure 2** Shows the percentage change in nucleosome concentration from the control following coincubation of the bladder carcinoma cell line 5637 with TGF-β1 (to initiate apoptosis) and increasing concentrations of hCGβ (to negate the TGF-β1 effect while the apoptotic factor was still present). 00 contained no hCGβ or TGF-β1 and represents the control to which the other values were corrected and further indicated by the dashed line

TGF-β receptor binding, given that it requires a dimeric species to align the receptor subunits and initiate the apoptotic cascade. The marked similarity in topology may be enough to allow binding but not to activate second messengers in addition and thus prevent the action of the autocrine TGF-β regulation on cell growth. The results here do not provide the information to determine the route by which hCGβ is acting. Given the complexity of an apoptotic cascade any cross-talk occurring may do so at any number of locations following stimulation by hCGβ and warrants further investigation.

In conclusion it appears that the recently described growth effect attributed to ectopic free hCGβ production is not due to growth stimulation but an anti-apoptosis effect which may be brought about in an antagonistic manner via a known receptor or receptor-mediated cascade. Whatever the mode of action occurring here the evidence for hCGβ as a growth modulator is increasing and its involvement in an apoptotic cascade opens up a new field for investigation.

### ACKNOWLEDGEMENTS

This work has been funded by grants from the Joint Research Board of St. Bartholomews Hospital, London, the Vandervell Foundation, the Garfield Weston Foundation and the Sir Samuel Scott of Yew Trust.

### REFERENCES

Braunstein GD (1983) hCG expression in trophoblastic and nontrophoblastic tumors. In: *Oncodevelopmental Markers: Biologic, Diagnostic and Monitoring Aspects*, Fishman WH (ed), pp. 35. Academic Press: New York

Butler SA, Laidler P, Porter JR, Kicman AT, Chard T, Cowan DA and Iles RK (1999) The beta subunit of human chorionic gonadotrophin exists as a homodimer. *J Mol Endocrinol* **22**: 185–192

Cole LA, Wang Y, Elliot M, Latif M, Chambers JT, Chambers SK and Schwartz PE (1988). Urinary human chorionic gonadotrophin free β-subunit and core fragmant: a new marker of gynecological cancers. *Cancer Res* **48**: 1356–1360

Gillott DJ, Iles RK and Chard T (1996) The effects of βhCG on the in vitro growth of bladder cancer cells. *Br J Cancer* **73**: 323–326

Iles RK and Chard T (1989) Immunochemical analysis of the human chorionic gonadotrophin-like material secreted by 'normal' and neoplastic urothelial cells. *J Mol Endocrinol* **2**: 107–112

Iles RK Butler SA and Jacoby E (1999) Dimerisation of urinary β-core/hCGβcf: A cause of poor β-core assay performance in Downs syndrome screening studies. *Prenatal Diagnosis* **19**: 790–792

Lapthorn AJ, Harris DC, Littlejohn A, Lustbader JW, Canfield RE, Machin KJ, Morgan FJ and Isaacs NW (1994) Crystal structure of hCG. *Nature* **369**: 455–461

Lunardi-Iskandar Y, Bryant JL, Zeman RA, Lam VH, Samaniego F, Besnier JM, Hermans P, Thierry AR, Gill P and Gallo RC (1995). Tumorigenesis and metastasis of neoplastic Kaposi's sarcoma cell line in immunodeficient mice blocked by a human pregnancy hormone. *Nature* **375**: 64–68

Mosmann T (1983) Rapid colorimetric assay for cellular growth and survival: application to proliferation and cytotoxicity assays. *J Immunol Methods* **65**: 55–63

Pierce JG and Parsons TF (1981) Glycoprotein hormones: structure and function. *Ann Rev Biochem* **50**: 465–495

Sporn MB, Roberts AB, Wakefield LM and Assoian RK (1986) Transforming growth factor-β: biological function and chemical structure. *Science* **233**: 532–534

Sun PD and Davies DR (1995) The cystine-knot growth-factor superfamily. *Ann Rev Biophys Biomol Struct* **24**: 269–291

Twentyman PR and Luscome M (1987) A study of some variables in a tetrazolium dye (MTT) based assay for cell growth and chemosensitivity. *Br J Cancer* **56**: 279–285

*© 2000 Cancer Research Campaign*
